# Identification and characteristics of Testudinis Carapax et Plustrum based on fingerprint profiles of mitochondrial DNA constructed by species-specific PCR and random amplified polymorphic DNA

**DOI:** 10.1080/23802359.2018.1507631

**Published:** 2018-10-31

**Authors:** Mingcheng Li, Miao Wang, Yuqing Zhou, Zitong Li, Guangxin Yuan, Xuesong Wang, Wei Xia, Jiayu Chen

**Affiliations:** a School of Laboratory Medicine, Beihua University, Jilin, People’s Republic of China;; b School of Pharmacy, Beihua University, Jilin, People’s Republic of China;; c School of Medicine, Shaoxing University, Shaoxing, People’s Republic of China

**Keywords:** Testudinis Carapax et Plustrum, mtDNA, PCR fingerprint profiles, cytochrome b

## Abstract

Traditional use of Testudinis Carapax et Plustrum (TCP) as a medicine and health food has been widely reported. We compared two DNA fingerprint profiles of mitochondrial (mtDNA) from TCP based on species-specific PCR and random amplified polymorphic DNA (RAPD) to identify their authority. A series of sequences from cytochrome b (*Cyt* b) of *Chinemys reevesii* and their counterfeits were downloaded from the Genbank, and Premier 5.0 software was used to design a set of primers. A species-specific PCR and RAPD were undertaken to obtain the different DNA fingerprints respectively. The mtDNA was successfully extracted from all samples using the modified salting-out method. A relative molecular mass of 16.6 × 10^3^ bp was observed, and mtDNA was measured between 1.83 ± 0.02. Fragments of 78 bp were amplified from all the TCP samples tested (except adulterant animals) using species-specific PCR method. The RAPD showed different electrocardiogram between genuine and counterfeit tortoise shell goods along with stripe number and location. The salting-out method (as modified) was used to extract high-quality mtDNA from TCP. The species-specific PCR and RAPD assay proposed in this study could be used for quality control of TCP with more specificity, sensitivity, and applicability.

## Introduction

Testudinis Carapax et Plustrum (TCP) is the carapace and belly from *Chinemys reevesii*, also known as turtle version and turtle shell. With the benefits of kidney strength, yin and yang, nourishing the blood, TCP can be used for the treatment of yin deficiency, hot flashes, sweating, flatulence, vertigo, etc. There are abundant species of *Chinemys reevesii* in China, yet there are remain many counterfeit goods in addition to artificial *Chinemys reevesii* in the drug market. Currently, authentication of *Chinemys reevesii* includes mostly authentication of origin, traits, and physicochemical properties: systematic studies of *Chinemys reevesii* remain rare (Chinese Pharmacopoeia 2010 edition). Authentication of traits is based on mainly shape, color, odor, taste, texture, and other aspects. The commonest method is microscopic authentication, which allows observation of the internal structure and morphology of *Chinemys reevesii*. This method is fast and easy, but cannot reveal genetic information about a species on a cellular basis, and thus cannot accurately identify closely-related species. In terms of advances in molecular biotechnology in recent years, varieties of DNA manipulation techniques have been developed that can potentially be applied in Chinese medicine authentication ).

DNA fingerprinting is a method of using DNA to identify species at the molecular level. It has high specificity, is more scientific, accurate than various another method. Additionally, the technique of random amplified polymorphic DNA (RAPD) has a rich polymorphism, needs a lower dosage of DNA amplification results and no special primers (Deng et al. 2014).

In the present study, we modified salting-out method to extract the mtDNA from turtle shells and optimized species-specific PCR and RAPD methods. The DNA fingerprint profiles were identified as a reference for the authenticity of TCP.

## Materials and methods

### Sample collection

The experimental samples used in this experiment included eight batches of fresh and dry TCP samples, all of which were purchased from the Chinese Institute for the Control of Pharmaceutical and Biological Products. Counterfeit tortoises included six species of *Trachemys scripta*, *Indotestudo elongata*, *Manouria impression*, *Ocadia sinensis*, *Graptemys geographica*, and *Notochely splatynota*; eight batches of commercially available samples labeled A1–A8 (dry goods, purchased from different pharmacy in Jilin City). All samples were identified by the Chinese Food and Drug Supervision and Management of Jilin. All animals’ tissues in the study were approved by the Medicine Institutional Animal Care and Use Committee, Beihua University (ethical approval number: Protocol Number 2012-10-12).

### Extraction and detection of mtDNA from experimental samples

One gram of each fresh sample was cut into small pieces measuring 0.1 mm ×0.1 mm ×0.1 mm, and dry samples (mass ≧0.1g) were washed with 78% alcohol and with double distilled water three times, and then stored in a mortar. mtDNA of turtle shells was extracted using the modified salting-out method. The purity and concentration of DNA samples were calculated by their A260/A280 ratio as described previously (Li et al. 2016).

### Primer design and PCR amplification reaction

The entire sequences of the *Cyt* b gene of Chinese tortoise and six common counterfeit turtles were downloaded from Genbank. Primers capable of specifically amplifying the *Cyt b* fragment of the *Chinemys reevesii* were synthesized by Sangon Biotech (Shanghai) Co., Ltd as follows:

Upstream primer: 5′-CGAAAAACTCACCCAATAAAAATCA-3′,
Downstream primer: 5′-TCATCATGCAGAGATATTAGAGGGA-3′.

PCR amplification reaction was performed as described previously (Gao et al. 2016).

### RAPD amplification and detection

Random primers were purchased from Shanghai JieRui Biological Engineering Co., Ltd. 10-mer random primers are screened, and primer S6 (CCCGCTACAC) was selected. 25 μL reaction system of RAPD amplification system consisting of 2 × Taq PCR Master Mix 12.5 μL, 1.0 μL primer (12.5 μmol/L), DNA template 2.0 μL, and ddH_2_O 9.5 μL. PCR circulation parameters included pre-denaturation for 5 min (94 °C), denaturation for 80 s (94 °C), annealing for 1 min (36 °C), and extended for 2 min (72 °C), 40 cycles extended again after 10 min (72 °C).

## Results

### mtDNA purity and concentration

**Figure 1.**
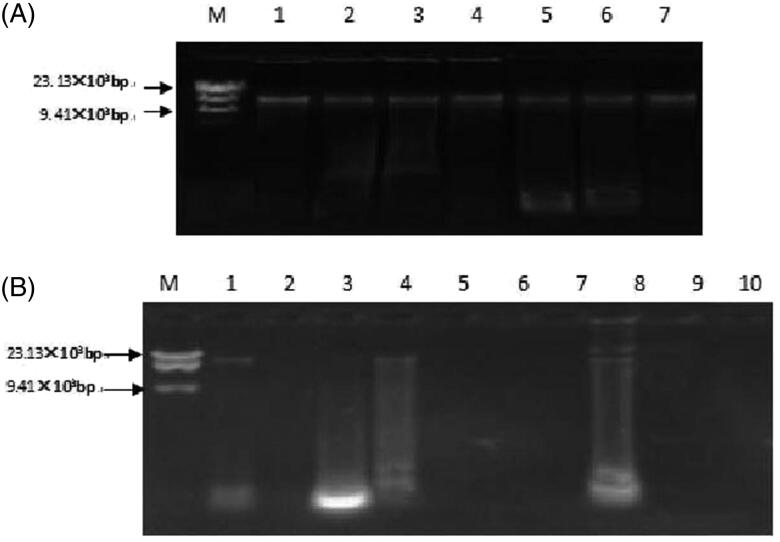
Agarose gel electrophoresis of mitochondrial DNA extracted from Testudinis Carapax et Plastrum, counterfeits and commercially available shells with salting-out method. (A) M: Marker; 1: Testudinis Carapax et Plastrum; 2: Ocadia sinensis; 3: Indotestudo elongate; 4: Graptemys geographica; 5: *Trachemys scripta*; 6: Manouria impressa; 7: Notochely splatynota. (B) M: marker; 1–8: Commercially available shells, A1–A8.

The purity of the mtDNA of fresh turtle shells extracted using modified salting-out was between 1.80 ± 0.12, which indicated that the extracted mtDNA were almost free of protein and alcohol, and the purity was high. Agarose gel electrophoresis showed that the extracted DNA bands were singular with a relative molecular mass of about 16.6 × 10^3^. The DNA purity of A1, A4, and A8 was between 1.80 ± 0.12, and the extracted DNA bands were the same as the single band. The purity of the remaining commercially available tortoise samples was less than 1.80 ± 0.12. Agarose gel electrophoresis showed DNA band dispersion or no bands (Figure 1(A,B)).

### PCR product on agarose gel electrophoresis

**Figure 2.**
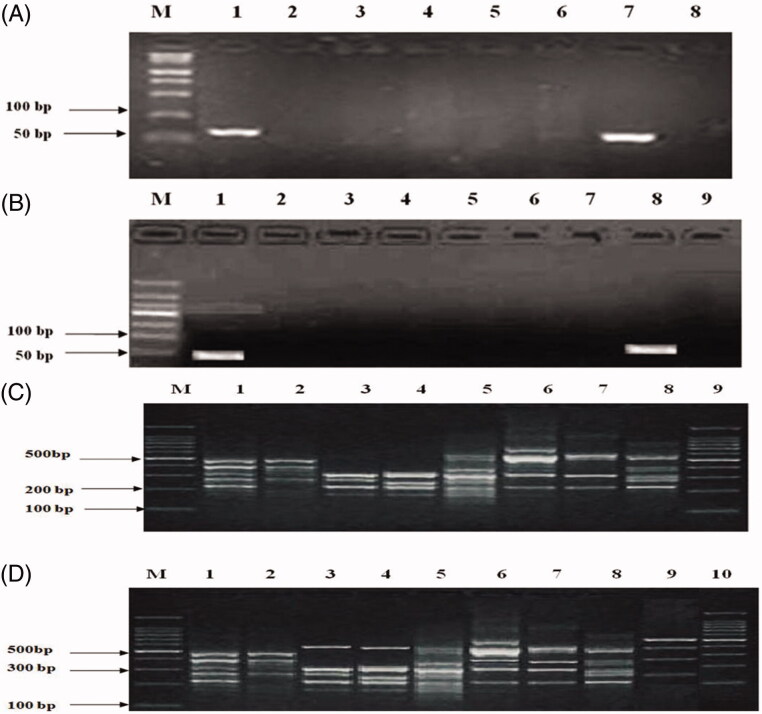
Comparison of PCR product of *Cyt* b gene segment and PCR-RAPD fingerprints from Testudinis Carapax et Plastrum, counterfeits and commercially available shells. (A) M: Marker; 1: Testudinis Carapax et Plastrum (fresh sample); 2: Ocadia sinensis; 3: Indotestudo elongate; 4: Graptemys geographica; 5: *Trachemys scripta*; 6: Manouria impressa; 7: Testudinis Carapax et Plastrum (dried sample); 8: Notochely splatynota. (B) M: marker; C: Testudinis Carapax et Plastrum; 1–8: Commercially available shells, A1–A8; 9: negative control. (C) M: Marker; 1: Testudinis Carapax et Plastrum (fresh sample); 2: Ocadia sinensis; 3: Indotestudo elongate; 4: Graptemys geographica; 5: *Trachemys scripta*; 6: Manouria impressa; 7: Notochely splatynota; 8: Testudinis Carapax et Plastrum (dried sample); 9: Marker. (D) M: marker; 1: Testudinis Carapax et Plastrum; 2–9: Commercially available shells, A1–A8; 10: Marker.Amplification of tortoise shell DNA-RAPD on agarose gel electrophoresis

The mtDNA extracted from genuine, counterfeit, and commercially available tortoise samples were amplified using PCR using the specific primers. There is a bright strip at 70–80 bp among genuine turtle shells including Chinese turtle, however, counterfeit turtles had no amplification strip (Figure 2(A)); the commercially available A8 tortoise shells showed only a bright band at 70–80 bp, whereas the remaining commercially available tortoise shells and negative controls no bands (Figure 2(B)).

### Amplification of tortoise shell DNA-RAPD on agarose gel electrophoresis

The electrophoretogram of RAPD with S6 (CCCGCTACAC) as the primer showed the same electrophoresis profiles between the two genuine shells. The fragments from 100 bp, 200 bp, 300 bp, 400 bp to 500 bp presented five characteristic bands, only with slight changes in brightness. Compared with the genuine turtle shells, there was an obvious difference among counterfeits electrophoresis, showing the band number and location difference. The four batches of commercially available samples, including A2, A3, and A4 (but not A8) showed the obvious difference compared with genuine shell electrophoresis, could thus be identified as counterfeit tortoise (Figure 2(C,D)).

## Discussion

In this experiment, we improved salting-out to extract the mtDNA from a turtle shell. The results showed that the modified salting-out method could obtain more complete, high-quality DNA in the extraction of fresh and dried turtle and comply with the requirements of PCR amplification reaction. The use of modified salting-out method can replace the traditional method of extracting genomic DNA, to avoid the operation of cumbersome, the use of toxic and harmful reagents. In the process of identification of genuine products and commercially available tortoise shells, we found that the amount of mtDNA extracts from dried turtle shells with modified salting-out method was small, and most of which were broken. This is due to the complexity of the processing of tortoise shells, which required high temperature, vinegar leaching process (Yip et al. 2007), resulting in the destruction of the integrity of the turtle DNA. The results demonstrated that the modified salting-out method to extract mtDNA did not affect subsequent PCR amplification.

The specific primers of this experiment are conserved sequences obtained from the highly stable *Cyt* b gene sequence. In the process of primer design, the design of the amplified product is 70–80 bp, which can avoid the error caused by the fragmentation of mtDNA. The experimental results show that only A8 can amplify the same result as the genuine turtle shell in the eight selected samples. The sample is genuine turtle shell, indicating that this method can be used for the sale of processed turtle shell medicine.

When the annealing temperature was 61 °C, only a genuine turtle shell showed a bright band at 78 bp. The results showed that the selected primers could distinguish the fresh samples from the genuine turtle shells and other common goods and solved the problem of authentic turtle shell and closest counterfeit identification. Another PCR-fingerprint based on RAPD analysis was also used to study genetic diversity of species in the absence of any molecular biology research tool with rich polymorphism, so it has been widely applied in authentication of species resources. This method also can distinguish between genuine and counterfeit tortoise shells goods.

Nowadays, a hybrid species of TCP is often seen on drug market in China. Because the wild species of TCP breed slowly at least for 10 years, counterfeits grow fast, the hybrid species show both the genuine and counterfeit tortoise shells profiles. A species-specific PCR failed in the identification of TCP only targeting the *Cyt b* gene, whereas the RAPD will solve the problem based on the whole genome.

A comparison with two methods showed that they both exhibited short-processing times; the DNA quality of the DNA template does not need to be high, and the technique is highly sensitive and repeatable. A rather trivial gene segment contains inheritance information for both inter- and intra-species evolution, therefore, both are very effective, reliable methods at molecular level for studying animal population genetics and evolutionary genetics, similar to the findings, which evinced a steady index capable of species identification and genetic marking reported by Bever and Yang (Bever et al. 2015).

A more convenient method for DNA molecular genetic marker identification is established. The proposed method was used to provide a strong basis for the identification of authenticity of *Chinemys reevesii*.
